# Meat Consumption in Sao Paulo – Brazil: Trend in the Last Decade

**DOI:** 10.1371/journal.pone.0096667

**Published:** 2014-05-02

**Authors:** Aline Martins de Carvalho, Chester Luiz Galvão César, Regina Mara Fisberg, Dirce Maria Marchioni

**Affiliations:** 1 Departament of Nutrition, School of Public Health – University of São Paulo, São Paulo, São Paulo, Brazil; 2 Departament of Epidemiology, School of Public Health – University of São Paulo, São Paulo, São Paulo, Brazil; 3 Departament of Nutrition, School of Public Health – University of São Paulo, São Paulo, São Paulo, Brazil; 4 Departament of Nutrition, School of Public Health – University of São Paulo, São Paulo, São Paulo, Brazil; Kagoshima University Graduate School of Medical and Dental Sciences, Japan

## Abstract

**Objective:**

To characterize trends in meat consumption, and verify the percentage of excessive red and processed meat consumption in the last decade in São Paulo, Brazil.

**Design:**

Cross-sectional weighted data from the Health Survey for São Paulo, conducted in São Paulo, Brazil among people aged 12 years and older.

**Setting:**

Diet was assessed by two 24-hour recalls in each survey. Usual meat consumption was estimated by Multiple Source Method. Wald tests were used to compare means across survey years. Data were collected from adolescents, adults, and elderly using a representative, complex, multistage probability-based survey in 2003 and in 2008 in São Paulo, southeast of Brazil.

**Subjects:**

2631 Brazilians were studied in 2003 and 1662 in 2008.

**Results:**

Daily mean of red and processed meat consumption was 100 g/day in 2003, and 113 g/day in 2008. Excessive red and processed meat consumption was observed in almost 75% of the subjects, especially among adolescents in both surveys. Beef represented the largest proportion of meat consumed, followed by poultry, pork and fish in both surveys.

**Conclusions:**

Daily red and processed meat consumption was higher in 2008 than in 2003, and almost the entire population consumed more than what is recommended by World Cancer Research Fund. Public health strategies are needed, in order to reduce red and processed meat consumption to the recommended amounts, for a healthy diet.

## Introduction

Meat is an important food item for human nutrition because it contains protein, minerals and vitamins [1], and also unsaturated and conjugated fatty acids that help prevent cardiovascular diseases [2]. Nevertheless, excessive meat consumption has been linked to chronic diseases. Some studies show the relationship between processed meat intake and cardiovascular diseases and diabetes [3], and other studies show the relationship between red and processed meat intake and colorectal cancer [4]–[8], weight gain [9] and high death risk [10]–[12]. Potential carcinogenic substances such as heterocyclic amines and polycyclic aromatic hydrocarbons (formed during the cooking process), high saturated fat, and cholesterol content can increase the risks for the diseases mentioned above. The addition of sodium and nitrite in processed meats also increase these risks [4]–[6].

Currently, the Brazilian Ministry of Health recommends one daily serving of meat (190 kcal) as part of a healthy diet [1]. The World Cancer Research Fund recommends a limited intake of up to 500 g of red or processed meat per week as a measure for cancer prevention [4]. However, many developed countries present over-consumption of meat [2], [13].

In Brazil, there are few representative studies about meat consumption and its health impacts. However, Brazil is the world’s second largest beef producer and the world’s largest beef exporter [14]. So, it is important to monitor the Brazilian population to promote healthy eating policies. The present study characterizes the trends in meat consumption and the percentage of excessive red and processed meat consumption in the last decade in São Paulo, Brazil.

## Materials and Methods

### Study population and data collection

The School of Public Health of the University of São Paulo Ethics Committee approved the project.

Data was derived from two independent cross-sectional representative, complex, multistage probability-based surveys titled Health Survey for São Paulo, conducted in São Paulo, Brazil in 2003 and in 2008 (ISA – Capital 2003 and ISA – Capital 2008). These surveys collected information on health, food intake, and life conditions of the population of São Paulo.

A two-stage cluster sampling was used: census tracts and household, in both surveys. In ISA – Capital 2003, in the first stage, the census tracts were drawn using probability proportional to the number of households in the PNAD 2002 (National Household Sample Survey 2002). In ISA- Capital 2008, in the first stage, the census tracts were drawn using probability proportional to the number of households in the PNAD 2005 (National Household Sample Survey 2005). In the second stage, the households were drawn using inverse probability of the number of households in each PNAD.

The draw was systematic, and the census tracts were stratified according to the percentage of heads of family with academic degrees into three categories (less than 5%; 5 to 25%; more than 25%).

Six study domains were defined in ISA – Capital 2003 and ISA – Capital 2008 by age groups and gender: women and men aged 13 to 19 years old (adolescents), women and men aged 20 to 59 years old (adults) and women and men aged 60 years old or over (elderly).

In 2003, it was estimated a minimum sample size of 420 interviews for each of the six domains based on a prevalence of 0.5 with a standard error of 0.06 at a 5% significance level and a design effect of 1.5. In ISA – Capital 2003, a total of 2515 individuals were selected, however the final sample comprised 2361 subjects (both males and females), 805 adolescents, 743 adults and 813 elderly. Of all selected participants, 6% (n = 153) refused to participate or could not be found at home, even after three visits made at different times (during weekdays and weekends).

In 2008, a new two-stage cluster sampling was used based on PNAD 2005, and the minimum of 300 interviews for each of the same six domains enabled estimation of a prevalence of 0.5 with a standard error of 0.07 at a 5% significance level and a design effect of 1.5. In ISA – Capital 2008, a total of 2691 individuals were selected, however the final sample comprised 1662 subjects (both males and females), 560 adolescents, 585 adults and 517 elderly. Of all selected participants, 38% (n = 1029) refused to participate or changed their address/telephone and could not be located or found at home, even after three visits made at different times (during weekdays and weekends). Even the loss was randomized among census tracts and socio demographic features, sampling weights were recalculated for each individual considering the sample design, the adjustment for non-response, and post-stratification adjustment for gender and age group, in order to equalize the socio demographic features of the sample. Other details on sampling are available elsewhere [15], [16].

Information on health and life condition was collected by a structured questionnaire administered during a household interview in 2003 and another in 2008. The questionnaires were structured for collecting demographic (age and gender) and socioeconomic (family income) data, and were administered by trained interviewers.

### Assessment of dietary intake

The dietary assessment consisted of two 24-hour dietary recalls (24HR) for each survey; they were collected over one year covering all weekdays, weekends and seasons [17].

In ISA – Capital 2003, the participation rate of two 24-hour recalls was 35%, and both 24HR was administered at households using Multiple Pass Method [18]. In ISA – Capital 2008, the participation rate of two 24-hour recalls was 50%, and the first 24HR was administered at households using Multiple Pass Method [18] and the second 24HR was administered by telephone using Automated Multiple Pass Method [19]. The telephone calls were made to the participants home or their mobile phone. These methods are structured in five steps: 1) quick list, that participants list all the foods and beverages consumed uninterruptedly; 2) forgotten list, that participants are asked about commonly forgotten foods consumed, such as candies, coffees and sodas; 3) time and location of food and beverage intake; 4) detailing cycle, that the way of preparation and amounts consumed are described; and 5) final review, that verifies whether a certain food consumed during the day was not previously recorded [18], [19].

The household measures reported in 24HR were converted into grams and milliliters according standard Brazilian references, that measure many foods in precision balance [20], [21]. Recipes were broken down into ingredients to estimate the amount of meat in each preparation.

Data from the 24HR were entered into the Nutrition Data System for Research – NDSR (version 5.0, 2007, Nutrition Coordinating Center at the University of Minnesota, Minneapolis, MN, USA) [22] and were converted into energy and nutrients. We compared the American database for the nutrition facts (energy, protein, carbohydrate and lipid) from the NDSR with the Brazilian nutrition facts database. We only considered the foods from the NDSR that were similar (between 0.8 until 1.2 times) to Brazilian nutrition facts in terms of energy and macronutrients.

The meats of the diet were classified according to origin: beef, pork, poultry and fish; and processing: processed meat (cured, salted, smoked or containing chemical preservatives); no processed red meat (beef and pork), no processed white meat (poultry and fish).

The World Cancer Research Fund [4] maximum recommendation intake of 500 g of red and processed meat per week (corresponding to mean of 71.4 g red and processed meat per day) was the cut-off point to estimate excessive red and processed meat consumption.

### Statistical Analysis

In both surveys, the second 24HR was used to remove within-person variation that would otherwise inflate the distribution thereby distorting the percentiles [23]. This adjustment was made by the Multiple Source Method (MSM), which requires that at least one participant provides both 24HR. However, a high participation rate of two 24HR (around 40%) leads more precise estimates [24].

The MSM is a statistical modeling technique which calculates usual dietary intake in three steps [25], [26]. In the first, the probability of eating the food on a random day for each individual was estimated by a logistic regression model. Secondly, the usual amount of food intake is estimated by a linear regression model. Finally, the resulting numbers from step one and two are multiplied by each other to estimate the usual daily intake for each individual. The models were performed separately by gender, furthermore age group and date of interview were included as model covariates in logistic and linear regressions to estimate probability of eating the meat and usual amount of meat.

All participants were considered meat consumers in MSM, because the technique could modify the first percentiles of distribution and it does not modify mean of usual intake of meat [27].

Mean values and standard errors were calculated considering the predicted usual intake distribution by MSM. The normality was verified by skewness and kurtosis normality test. Differences between means were analyzed using the Wald test, which calculates point estimates using F-statistics and considers the weights from complex samples.

The analyses were conducted using weighting variables (primary sampling unit, stratum and sampling weight) to account for the complex survey design. Data were analyzed separately by gender, *per capita* family income and age group. For all analyses, STATA statistical software package version 10 [28] was used and a p<0.05 was considered statistically significant.

## Results

The unweighted sample comprised a total of 4023 people from both data collection, 49% were male in 2003 and 44% were male in 2008; mean age was 41±24 years in 2003 and 37±26 years in 2008; mean *per capita* family income was U$167 in 2003 and U$383 in 2008. The proportions of men and women were the same in each age group and in each tertile of *per capita* income in both surveys. The population in the study showed an increase in consumption of the different types of meat from 2003 to 2008. Women, elderly and low-income groups were the only ones who did not show higher red meat consumption in 2008 than in 2003. There was an increase in white and processed meat intake for the entire population (Table 1). Adolescents and men also showed an increase in beef intake, while the elderly showed a decrease. Fish consumption rose for all groups but for the elderly and individuals with intermediary income. Intake of pork did not increase among the elderly and individuals with low and intermediary income. Poultry consumption increased for the entire population (Table 2).

**Table 1:** Dietary intake of total meat, red meat, processed and white meat (g/day) according to age, gender, *per capita* family income and year studied. São Paulo. 2013.

	Total meat (g/day)^a^						Red meat (g/day)^b^				White meat (g/day)^c^				Processed meat (g/day)^d^			
	2003			2008			2003		2008		2003		2008		2003		2008	
	n	Mean	SE	n	Mean	SE	Mean	SE	Mean	SE	Mean	SE	Mean	SE	Mean	SE	Mean	SE
Age group																		
adolescent	805	142.7	1.6	560	178.6*	5.2	74.8	1.5	92.6*	3.5	35.7	1.0	45.3*	1.2	32.9	0.8	42.3*	1.3
adult	743	136.5	1.7	585	167.7*	4.4	72.7	1.2	81.4*	2.5	37.7	1.0	52.6*	1,.7	28,.1	1.1	32.8*	0.9
elderly	813	121.3	1.3	517	124.9	2.8	65.5	1.3	62.0	2.3	37.8	0.9	45.8*	1.1	19.8	0.7	23.1*	0.9
Gender																		
male	1155	164.7	0.99	722	200.2*	6.0	89.0	1.3	104.1*	3.2	42.6	1.3	57.2*	2.1	33.7	1.1	39.6*	1.0
female	1206	112.2	1.4	940	130.6*	2.3	58.5	1.1	59.4	1.4	33.1	0.7	44.8*	1.2	23.2	0.8	26.8*	0.9
Per capita income																		
1 tertile	664	130.2	2.6	531	150.0*	5.2	68.3	1.6	74.1	3.8	34.2	1.3	48.5*	1.9	26.3	1.5	31.7*	1.3
2 tertile	745	138.4	2.1	582	163.8*	6.1	72.4	1.6	83.1*	3.2	38.0	1.5	49.5*	2.3	29.0	1.3	32.4*	1.3
3 tertile	821	138.9	1.7	549	171.5*	4.9	75.8	1.6	82.2*	2.9	39.5	1.2	53.0*	1.9	28.4	0.9	33.8*	1.1
TOTAL	2361	135.8	1.25	1662	163.2*	3.5	72.2	0.9	80.3*	1.8	37.4	0.8	50.6*	1.2	27.9	0.8	32.8*	0.7

*Wald test (difference between consumption in 2003 and consumption in 2008, considering p<0.05 significant).

a Total meat: all types of meat consumed.

b Red meat: unprocessed beef and pork.

c White meat: unprocessed fish and poultry.

d Processed meat: cured, salted, and smoked meats or meats containing preservatives.

SE: standard error.

**Table 2:** Beef, pork, poultry and fish intake (g/day) according to age group, gender, per capita family income and year studied. São Paulo. 2013.

	Beef (g/day)						Pork (g/day)				Poultry (g/day)				Fish (g/day)				
	2003			2008			2003		2008		2003		2008		2003		2008		
	N	Mean	SE	N	Mean	SE	Mean	SE	Mean	SE	mean	SE	mean	SE	mean	SE	mean	SE	
Age group																			
adolescent	805	74.2	1.8	560	89.3*	2.7	27.1	1.1	41.4*	2.6	33.0	0.9	41.3*	1.0	6.7	0.8	11.6*	0.7	
adult	743	72.4	1.4	585	76.7	2	24.6	1.4	30.7*	1.7	32.5	1.0	42.5*	1.2	10.0	1.0	14.8*	0.9	
elderly	813	63.1	1.2	517	57.6*	1.7	18.2	1.0	20.3	1.6	29.3	0.8	37.8*	0.9	11.2	0.9	12.4	0.6	
Gender																			
male	1155	87.4	1.8	722	96.5*	2.6	30.7	1.7	39.3*	2.2	35.0	1.2	44.3*	1.5	13.3	1.5	17.5*	1.2	
female	1206	58.8	1.1	940	57.5	1.2	19	1.0	23.2*	1.1	29.9	0.7	39.3*	0.9	6.6	0.4	10.9*	0.7	
Per capita income																			
1 tertile	664	68.4	1.9	531	70.9	3.3	22.8	2.2	28	2.3	30.4	1.1	40.9*	1.3	7.4	0.9	12.6*	1.3	
2 tertile	745	72.2	1.9	582	78	2.9	26.4	1.6	29.4	2.5	32.5	1.3	41.6*	2.2	9.8	1.7	12.9	1	
3 tertile	821	74.1	1.7	549	77.2	2.2	24.1	1.3	33.7*	2.4	34.1	1.4	42.2*	1.3	10.6	0.8	15.9*	1.1	
TOTAL	2361	71.7	1.1	1662	75.8*	1.4	24.3	1.1	30.8*	1.3	32.2	0.8	41.7*	0.8	9.6	0.7	14.0*	0.7	

*Wald test (difference between consumption in 2003 and consumption in 2008, considering p<0.05 significant).

SE: standard error.

There was a 20% increase in average meat consumption, with a greater increase in white meat intake (35%) and lower increase in red meat intake (11%). Processed meat intake also increased during the periods studied (20%), especially among adolescents (29%). As for the origin of the meat, the increase in consumption was greater for fish (46%), followed by poultry (30%), pork (30%) and beef (1%).

Among the most frequently consumed processed meats by the citizens of São Paulo, sausages and frankfurter represented 60% of the processed meat intake in both the periods studied, and were followed by ham, industrialized breaded chicken and mortadella (data not shown).

In 2003, 72% of residents of the city of São Paulo exceeded red and processed meat intake recommendations from the WCRF, and in 2008, this number was 74% (a non significant variation). The proportion of individuals from the different age groups and genders that exceeded red and processed meat intake recommendations was the same in both studies (data not shown).

## Discussion

We observed a significant increase in meat consumption in the city of São Paulo from 2003 to 2008, especially in total meat, poultry, white and processed meat intakes that increased regardless of gender and *per capita* family income.

It is known that there is an increasing tendency of poultry production in Brazil, and this might be a good factor once poultry has leaner meat and therefore can improve diet quality [1], [29]. However, there is also an increased tendency in producing processed meats that have higher fat contents, apart from having potentially carcinogenic substances such as nitrites and nitrates, and sodium [1], [4].

These increasing intake tendencies shown in this study can be explained due to the low prices of poultry and processed meats [30], the increasing poultry and processed meat production, the Brazil economic stability over the past few years, what increased the population purchasing power [31], and also because meat is a typical food within Brazilian eating habits and desired by most of the population.

We observed that red meat and beef did not show a significant consumption increase in any of the analyzed categories, but were the most consumed in both periods. Fish, on the contrary, was the least consumed by the city's residents. We found similar data to the tendency observed in São Paulo in the last Brazilian Household Budget Survey (1987–1988; 1995–1996; 2002–2003; 2008–2009). Beef had the greatest energy contribution in the Brazilian population diet in the periods studied, but underwent a decline over the last years. Meanwhile, poultry consumption showed a progressive increase throughout the whole period (150%). Fish intake had low and constant contribution, less than 1% [32], [33].

In regards to meat consumption around the world, available data from the Food and Agriculture Organization show an increasing number in daily total meat intake in developed countries such as the US [13] and the European Nations [2]. We also noticed an increase in white meat intake (from 25 g to 55 g/day) and decrease in red meat intake (from 105 g to 85 g/day) in the US from 1999 to 2007 [13].

Total meat and red meat intake in São Paulo proved to be higher or similar than those found in developed countries such as the US [13], Germany, Ireland, Spain and the Netherlands [2]. That is, the citizens of São Paulo consumed more red meat than those in developed countries. For processed meat, the intake was the same as that of the US [13] and greater than that of Ireland, Greece and Italy [4].

Meat provides an important source of protein and micronutrients for humans, however excessive red and processed meat consumption is known to be associated to an increase in risk of colon and rectal cancer [4]. It is known that the intake of 50 g of processed meat a day increases the risk of CVD by 42%, and of diabetes by 19% in the US [3]. In our study, we noticed that almost 75% of the population showed excessive red and processed meat intake, what may increase the prevalence of these diseases in the city of São Paulo. Red and processed meat intake among adolescents was also high, what may contribute to increased risk of cancer later in life. An American cohort study showed that processed meat intake during adolescence increased the risk of colon and rectal cancer [34] in 25% among adolescents with high consumption.

Cancer incidence has been increasing significantly for the past decades and was one of the main causes of death from 1980 to 2010, in the city of São Paulo. Colon and rectal cancer is the third most frequent type of cancer among men and women. From 1997 to 2008 almost 17.0 thousand new cases were diagnosed in men; and at the same time there were 18.5 thousand new cases among women. The incidence of this type of cancer increased in 24% and 39% among men and women, respectively, from 2003 to 2008 [35]. It is well known that diet has an important role in preventing and causing this type of cancer and there is convincing evidence of the relationship among red and processed meats increase in risks of colon and rectal cancer [4].

### Limitation

The ISA – Capital is a cross-sectional study in which we cannot determine causality of events, but by using a probability sample and being a population-based study, results can be extrapolated to the total population of São Paulo, the biggest city of Brazil.

The loss of subjects in ISA – Capital 2008 was high, however sampling weights were recalculated for each individual, in order to equalize the socio demographic features of the sample and to produce validated results.

## Conclusions

Data from the present study allowed us to conclude that red and processed meat intake was excessive in almost the entire population studied, and there was a higher consumption of meats, particularly poultry and processed meats, in 2008 than in 2003. Therefore, developing public health actions is critical for health promotion and health food choices.
